# Association of domain-specific physical activity and cardiorespiratory fitness with all-cause and cause-specific mortality in two population-based cohort studies

**DOI:** 10.1038/s41598-018-34468-7

**Published:** 2018-10-30

**Authors:** Martin Bahls, Stefan Groß, Sebastian E. Baumeister, Henry Völzke, Sven Gläser, Ralf Ewert, Marcello R. P. Markus, Daniel Medenwald, Alexander Kluttig, Stephan B. Felix, Marcus Dörr

**Affiliations:** 1 Department of Internal Medicine B, University Medicine Greifswald, Greifswald, Germany; 2 DZHK (German Centre for Cardiovascular Research), Partner Site Greifswald, Greifswald, Germany; 3 Chair of Epidemiology, Ludwig-Maximilians-Universität München, UNIKA-T Augsburg, Augsburg, Germany; 4 Institute for Community Medicine, University of Greifswald, Greifswald, Germany; 5 Vivantes Klinikum Spandau, Berlin, Germany; 6 Martin-Luther-Universität Halle-Wittenberg Institute of Medical Epidemiology, Biometry and Informatics, Halle (Saale), Germany

## Abstract

Physical activity (PA) reduces the risk for mortality. Whether the beneficial effects of PA are domain specific is unclear. We associated leisure time (LTPA), sports (SPA) and work (WPA) related PA and cardiorespiratory fitness (CRF) with all-cause mortality in two German population-based cohorts. We used data of the Study of Health in Pomerania (SHIP, n = 2,935, median age 53; 48% male) and the Cardiovascular Disease, Living and Ageing in Halle study (CARLA, n = 1,776, median age 64 and 54% male). Mortality was determined after a median follow-up of 8.2 years in SHIP (n = 332) and 11.5 years in CARLA (n = 409). LTPA (SHIP: hazard ratio [HR] per standard deviation [SD] 0.82 95%-CI 0.73 to 0.91 and CARLA: HR per SD 0.70: 95%-CI 0.59 to 0.82) and SPA (SHIP: HR per SD 0.80 95%-CI 0.71 to 0.91 and CARLA: HR per SD 0.70 95%-CI 0.60 to 0.82) but not WPA were inversely associated with all-cause mortality. In a subsample CRF was inversely related to mortality and positively to LTPA and sports SPA. No association was found for WPA. Our results may suggest that the inverse association between PA and mortality are partly influenced by higher CRF.

## Introduction

Physical activity is the hallmark of a healthy lifestyle and is essential for the prevention of non-communicable diseases^1,2^. Epidemiological studies have demonstrated an inverse relationship between physical activity and all-cause mortality^3–5^. Further, regular physical activity reduces the risk for cardiovascular disease (CVD)^6,7^ and cancer^8^. The well-established and undisputed beneficial effects of regular physical activity are exemplified by the current guidelines, which recommend at least 150 minutes of moderate intensity exercise per week^9–11^.

Physical activity can take place in different domains. For example, it might be related to walking during leisure time (LTPA), exercising in a sport setting (SPA) or may be work related (WPA). A recent large meta-analysis which included more than 1.3 million individuals concluded that independent of domain, physical activity reduces the relative risk for all-cause mortality^12^. In contrast, a current investigation exploring the association of WPA on risk for ischemic heart disease in 12,093 nurses with a follow-up of 14 years detected a U-shaped relationship between risk for ischemic heart disease and WPA^13^. Therefore, if physical activity has beneficial effects independent of the setting in which it occurs is currently under debate.

We investigated the relationship between physical activity in three different settings (i.e. LTPA, SPA and WPA) with all-cause, CVD and cancer mortality in two independent German population-based studies. In addition, in a subset of individuals we assessed the relation between maximal oxygen consumption (VO_2_peak), maximal oxygen consumption normalized for body weight (VO_2_peak/kg), oxygen consumption at the anaerobic threshold (VO_2_@AT) and maximal workload (Wmax) assessed by standardized cardiopulmonary exercise testing (CPET) with all-cause, CVD and cancer mortality as well as the different physical activity domains.

## Results

### Baseline characteristics

A total of 2,935 SHIP participants with a median age of 53 years (range: 25–86) with 48% men were included in the analysis. The baseline characteristics are provided in Table 1 (additional information on cause specific mortality is provided in Suppl. Tables 1 and 2 for CVD and cancer mortality, respectively). Subjects who died during follow-up (n = 332) were older, had lower income, less education and a higher BMI. Further, these individuals had lower cardio-respiratory fitness as well as less LTPA and SPA but more WPA. SHIP participants with higher LTPA tended to be more often female, had a lower BMI, smoked less and received a higher amount of schooling. Individuals with a higher SPA were younger, had a lower BMI, a higher income and more schooling as well as consumed more alcohol. High WPA was related to a younger age, lower income, and less schooling (Suppl. Table 3).

**Table 1:** Population description of the SHIP study participants.

Parameter	Survivors subgroup (n = 2603)	All-cause mortality subgroup (n = 332)	All subjects (n = 2935)
Age (years)	51 (39; 62)	72.5 (64; 79)	53 (41; 65)
Income (€)	1100 (895; 1525)	1096 (778; 1450)	1100 (778; 1525)
Alcohol consumption (ml/day)	4.0 (1.3; 12.3)	1.96 (0; 10.2)	3.92 (0.99; 12.08)
School education, % <10 years	32.9	75.9	37.7
BMI (kg/m2)	27 (24; 31)	29 (26; 33)	27 (24; 31)
VO_2_peak (ml/min)	1900 (1548; 2400)	1560 (1300; 1879)	1883 (1521; 2371)
VO_2_@AT (ml/min)	1082 (900; 1300)	1000 (840; 1150)	1050 (900; 1300)
max. Watt	148 (116; 196)	116 (84; 148)	148 (116; 180)
LTPA	3.25 (2.75; 3.75)	3.0 (2.5; 3.5)	3.25 (2.75; 3.75)
SPA	2.25 (2.0; 3.0)	2 (1.75; 2.5)	2.25 (2.00; 2.75)
WPA	2.85 (2.14; 3.57)	2.93 (2.43; 3.71)	2.86 (2.14; 3.57)
Sex (%male)	45.2	66.9	47.6
**Smoke status**			
never	27.3	21.4	26.6
former	43.1	30.4	41.5
current	29.7	48.2	31.8

SHIP: Study of Health in Pomerania. BMI: body mass index. VO_2_: oxygen uptake. AT: aerobic threshold LTPA: leisure time physical activity. SPA: sports-related physical activity. WPA: work-related physical activity. All values are given as median (25^th^ and 75^th^ percentile) or percentage for categorical variables.

The baseline characteristics for CARLA are provided in Table 2 (additional information on cause specific mortality is provided in Suppl. Tables 4 and 5 for CVD and cancer mortality, respectively). Overall 1,779 CARLA participants of whom 417 died were included in this analysis. Their median age was 64 (range: 45–83) years and 54% were men. Subjects who died were older and mostly male, had less schooling, less LTPA as well as SPA but more WPA. CARLA participants with high LTPA tended to be more often male, received more years of education and consumed more alcohol. CARLA participants with higher SPA were younger, received more years of education, had a lower BMI and consumed more alcohol. A high WPA in CARLA was related to being more often male, lower income and more alcohol consumption (Suppl. Table 6).

**Table 2:** Population description of the CARLA study participants.

Parameter	Survivors only (n = 1362)	All-cause mortality subgroup (n = 417)	All subjects (n = 1779)
Age (years)	62 (54; 69)	75 (68; 79)	64 (56; 73)
Income (€)	1750 (1250; 2250)	1750 (1250; 2250)	1750 (1250; 2250)
Alcohol consumption (ml/day)	5.0 (0.0; 16.8)	5.0 (0.0; 18.5)	5.0 (0.0; 17.4)
School education, % < 10 years	34.2	61.9	40.7
BMI (kg/m2)	27.8 (25.0; 30.9)	28.1 (25.5; 31.4)	27.9 (25.1; 31.0)
LTPA	3.3 (2.8; 3.5)	3.0 (2.5; 3.5)	3.3 (2.8; 3.5)
SPA	2.3 (1.8; 3.0)	2.0 (1.8; 2.6)	2.3 (1.8; 3.0)
WPA	2.6 (2.1; 3.4)	2.8 (2.3; 3.5)	2.6 (2.1; 3.4)
Sex (%male)	49.8	69.3	54.4
**Smoke status**			
Never	47.9	35.1	44.9
Former	32.6	46.1	35.8
Current	19.5	18.7	19.3

(CARLA: Cardiovascular Disease, Living and Ageing in Halle study. BMI: body mass index. LTPA: leisure time physical activity. SPA: sports-related physical activity. WPA: work-related physical activity. All values are given as median (25^th^ and 75^th^ percentile) or percentages for categorical variables).

#### Relationships among Baecke indices

LTPA and SPA were significantly correlated (r = 0.25, p = <0.0001). However, WPA was not correlated with LTPA (r = 0.02, p = 0.53) or SPA (r = −0.04, p = 0.17).

#### Relationship between physical activity and mortality

Table 3 shows the association between LTPA, SPA and WPA with all-cause, CVD and cancer mortality in SHIP. A one standard deviation (SD) increase in LTPA was related to 18% less risk of all-cause and a 17% lower risk of CVD mortality. There was no association with cancer mortality. A one SD increase in SPA was associated with a 20% lower all-cause and a 20% lower CVD but not with cancer mortality. WPA was not related to mortality.

**Table 3:** Hazards ratios for the association between physical activity domains with all-cause and cause-specific mortality in SHIP.

	All-cause mortality			CVD mortality			Cancer mortality		
	Cases/PY	incidence per 10000 PY	HR (95% CI)	Cases/PY	Incidence per 10000 PY	HR (95% CI)	Cases/PY	Incidence per 10000 PY	HR (95% CI)
LTPA	332/23,530	141	**0.82 (0.73 to 0.91)**	106/23,373	45	**0.83 (0.67 to 1.00)**	84/23,373	36	1.02 (0.82 to 1.28)
SPA	321/23,365	137	**0.80 (0.71 to 0.91)**	106/23,365	45	**0.80 (0.64 to 1.00)**	84/23,365	36	0.80 (0.63 to 1.03)
WPA	34/11,740	29	1.03 (0.71 to 1.49)	6/11,740	5	1.29 (0.51 to 3.28)	13/11,741	11	0.82 (0.47 to 1.46)

(SD, standard deviation. HR, hazards ratio. CI, confidence interval. PY = person years. All models included age, sex, years of schooling, income, smoking, body mass index in addition to the exposure variables. One Cox regression for a one SD increase in LTPA, SPA and WPA was run for each exposure-outcome combination).

Table 4 shows the association between LTPA, SPA and WPA with all-cause, CVD and cancer mortality in CARLA. In this independent study, a one SD increase in LTPA was related to a 30% lower all-cause, 38% lower CVD and 30% lower cancer mortality. A one SD increase in SPA was associated with 30% lower all-cause and 45% lower CVD mortality risk, but was not related to cancer mortality. Similar to SHIP, WPA was not associated with mortality.

**Table 4:** Hazards ratios for the association between physical activity domains with all-cause and cause-specific mortality in CARLA. All models included age, sex, years of schooling, income, smoking, body mass index in addition to the exposure variables.

	All-cause mortality			CVD mortality			Cancer mortality		
	Cases/PY	Incidence per 10000 PY	HR (95% CI)	Cases/PY	Incidence per 10000 PY	HR (95% CI)	Cases/PY	Incidence per 10000 PY	HR (95% CI)
LTPA	409/18,839	217.1	**0.70 (0.59, 0.82)**	153/18,839	81.2	**0.62 (0.47, 0.81)**	133/18,839	70.6	**0.70 (0.52, 0.92)**
SPA	405/18,812	215.3	**0.70 (0.60, 0.82)**	151/18,812	80.3	**0.55 (0.42, 0.72)**	132/18,812	70.2	0.84 (0.64, 1.09)
WPA	19/4,538	41.9	1.13 (0.56, 2.27)	6/4,538	13.2	2.24 (0.57, 8.84)	7/4,538	15.2	1.42 (0.45, 4.46)

One Cox regression for a one SD increase in LTPA, SPA and WPA was run for each exposure-outcome combination. (SD, standard deviation. HR, hazards ratio. CI, confidence interval. PY = person years).

#### Relationship between CRF and mortality

Table 5 shows the association between VO_2_peak, VO_2_peak/kg, VO_2_@AT and Wmax with all-cause, CVD and cancer mortality. In this analysis, a one SD increase in VO_2_peak was related to 44% lower all-cause, 67% lower CVD and 61% lower cancer mortality risk. When adjusted for body weight, a one SD increase in VO_2_peak/kg was associated with 53% lower risk for all-cause, 74% lower risk for CVD and 50% lower risk for cancer mortality. A one SD increase in VO_2_@AT was related to a 26% lower all cause and 51% lower cancer mortality but not CVD mortality risk. A one SD increase in Wmax was associated with a 62% lower all-cause, 71% lower CVD and a 65% lower cancer mortality risk.

**Table 5:** Cox regression model for the association between measures of cardiorespiratory fitness with all-cause mortality and cause-specific mortality in SHIP-1.

	All-cause mortality	CVD Mortality	Cancer Mortality
	HR (95% CI)	HR (95% CI)	HR (95% CI)
Cases/Person years	80/12,416	18/12416	30/12416
Incidence rate per 10000 PY	64	14	24
VO_2_peak (per SD)	**0.43 (0.28 to 0.66)**	**0.33 (0.12 to 0.90)**	**0.39 (0.20 to 0.76)**
VO2peak/kg (per SD)	**0.47 (0.33 to 0.67)**	**0.26 (0.10 to 0.64)**	**0.50 (0.29 to 0.89)**
VO_2_@AT (per SD)	0.74 (0.67 to 1.06)	1.00 (0.45 to 2.20)	**0.49 (0.27 to 0.88)**
Wmax (per SD)	**0.38 (0.25 to 0.57)**	**0.29 (0.12 to 0.75)**	**0.35 (0.18 to 0.66)**

All models included age, sex, years of schooling, income, smoking, body mass index in addition to the exposure variables. One Cox regression with one SD increase in CPET parameters was run for each exposure-outcome combination. (SD, standard deviation. HR, hazard ratio. CI, confidence interval. VO_2_peak: peak oxygen consumption. VO_2_peak/kg: peak oxygen consumption per kilogram body weight. VO_2_@AT: oxygen consumption at the aerobic threshold. Wmax: maximal workload).

#### Relationship between Baecke Scores and CRF

A one point increase in LTPA was related with higher VO_2_ peak (linear regression coefficient [β]: 129; 95%-confidence interval [CI]: 99 to 159 ml/min) and VO_2_/kg (β: 1.61; 95%-CI: 1.25 to 1.97 ml/min/kg). Similar results were observed for SPA. Specifically, a one point increase in SPA was associated with a higher VO_2_peak (β: 198; 95%-CI: 172 to 224 ml/min) and VO_2_peak/kg (β: 2.58; 95%-CI: 2.26 to 2.89 ml/min/kg). However, a one point increase in WPA was related with lower VO_2_peak (β: −27; 95%-CI: −10 to −44 ml/min) and VO_2_/kg (β: −0.40; 95%-CI: −0.82 to 0.01 ml/min/kg).

#### Sensitivity analysis for the relationship between physical activity and mortality in subjects who participated in CPET

Suppl. Table 7 shows the association between LTPA, SPA and WPA with all-cause, CVD as well as cancer mortality in SHIP participants who participated in CPET. Within this subgroup, a one SD increase in SPA was associated with a 35% lower risk for cancer mortality. LTPA and WPA were not associated with mortality.

## Discussion

Despite previous reports about the benefits of physical activity and exercise with regards to lower mortality, the relationship between different physical activity domains and mortality still remains unclear. We used two independent population-based cohort studies from two German regions to explore the relation between LTPA, SPA and WPA with mortality. In these independent studies, LTPA and SPA were inversely associated with all-cause and CVD mortality. WPA, on the other hand, had no protective effect. Interestingly, we found heterogeneous associations between LTPA and SPA with regards to cancer mortality. Only LTPA in CARLA was associated with lower cancer mortality. One may speculate that reasons for this discrepancy are related to the higher age or cancer incidence rates of CARLA compared to SHIP participants. Importantly, WPA was unrelated with any type of mortality in both cohorts. The novelty of this investigation is that in addition to providing information about the relationship between domain-specific physical activity based on a well-established questionnaire, we also used CPET to quantitatively assess cardio-respiratory fitness. While LTPA and SPA were associated with higher levels of fitness, there was no such association for WPA. Therefore, our results support the notion that the reduced mortality risk for individuals who are physically active during their leisure time or in a sports-related setting may be driven by increased cardio-respiratory fitness.

There is overwhelming consensus about the health benefits of LTPA and SPA. For example, in a large population-based sample from the U.K. (EPIC-Norfolk)^14^ any type of physical activity was associated with a significantly reduced risk for all-cause and CVD mortality compared to an inactive lifestyle. A second study of that same cohort^15^ also reported inverse relations between LTPA and SPA with all-cause and CVD mortality. In the population-based “Cooperative Health Research in the Region of Augsburg” (KORA) study^16^, LTPA was inversely associated with all-cause, CVD and cancer mortality. In a large sample from Switzerland self-reported physical activity^17^ was inversely associated with all-cause and CVD mortality in a dose-response relationship. Our results agree with these previous studies in that LTPA and SPA were related to a lower risk for all-cause mortality.

The relationship between WPA and mortality is still a conundrum. The EPIC-Norfolk study reported no relation between WPA and all-cause and CVD mortality^15^. The above mentioned study from Switzerland supports these findings and reported no relation between WPA and mortality^17^. Likewise, in an elderly Taiwanese population (aged 65 and older) WPA was not associated with all-cause mortality^18^, while a large study from Israel (Cardiovascular Occupational Risk Factor Determination in Israel Study) reported that high levels of WPA were related to increased CVD events^19^. Our results also suggest no relation between WPA and all-cause, CVD as well as cancer mortality. However, in KORA WPA was associated with a lower risk for all-cause and CVD but not cancer mortality^16^. These findings are partly supported by a meta-analysis which used data from six studies with more than 83,000 participants^12^. Even though they report a significantly inverse association between WPA and all-cause mortality, the authors also found a significant amount of heterogeneity between the analyzed studies (represented by an I^2^ of 87.6). We believe that these different results may potentially be explained by the questions used to assess WPA. For example, in the Taiwanese study WPA was assessed by asking the subjects if their job involved standing or walking^18^. The “Tecumseh Occupational Activity Questionnaire” used by EPIC, on the other hand, asked more detailed questions regarding the number of stairs climbed or any hard labor related to WPA. Additionally, WPA may be strenuous but not be comparable to LTPA and SPA with regards to its training effect on the musculoskeletal and cardiovascular system. The majority of work related questions of the Baecke questionnaire focus on strenuous activities (e.g. heavy lifting, sweating and tiredness after work) which may not necessarily be related to an improved aerobic exercise capacity. This is supported by the relationship between cardio-respiratory fitness and WPA in our study which even suggested an inverse association. Overall, the association of WPA with mortality requires further research and should employ methods which objectively and quantitatively not just measure activity but also physiological responses like breathing patterns, heart rate and sympathetic nervous system activation.

Our findings may be interpreted as contradicting compared to the classical studies from the 1950^th^ by Morris *et al*.^20,21^. They were the first to report that walking bus conductors and mail carriers had lower incidences of coronary heart disease compared to sedentary bus drivers and civil servants. The differences between theirs and our findings may be explained by the Baecke questionnaire. As mentioned above, WPA was assessed with questions that focus on work intensity. The bus conductors and mail carriers would have scored very low here, as their occupation includes mostly walking with a variable amount of stair climbing and light carrying. Therefore, the choice of the Baecke questionnaire may have biased our results. Nonetheless, our findings are in agreement with other large classical studies by Paffenbarger *et al*. who reported that longshoremen had similar death rates from coronary heart disease compared to sedentary controls^22^. Further, they reported that physical activity measured by weekly caloric expenditure was associated with lower incidence of myocardial infarction as well as all-cause and CVD mortality in Harvard alumni^23,24^. Specifically, these authors assessed LTPA by asking how many miles the study participants walked, climbed stairs as well as played light and vigorous sports. Our findings are also in agreement with a recent meta-analysis that even reported a trend towards an inverse relationship between WPA and mortality in 193,696 subjects^25^. We also found that LTPA and SPA are associated with a lower incidence of all-cause, CVD and cancer mortality. Overall, methodological differences need to be taken into consideration when assessing the association of domain specific physical activity with mortality.

Even though, one may speculate that increases in cardio-respiratory fitness are driven by higher levels of physical activity^26,27^, physical activity only explains 1% to 36% of the variance of cardio-respiratory fitness^28,29^. Hence, physical activity and cardio-respiratory fitness may both reduce mortality through different mechanisms. Studies which investigated the independent associations between cardio-respiratory fitness and self-reported physical activity with all-cause mortality had heterogeneous findings^30–33^. Some reported that physical activity is a significant predictor of mortality after adjusting for cardio-respiratory fitness^29,31,32^ whereas others have reported that physical activity is not associated with mortality after controlling for cardio-respiratory fitness^30^. Our results further spur this discussion by providing important information regarding domain-specific physical activity. Specifically, we report that LTPA and SPA are associated with higher cardio-respiratory fitness as well as lower mortality. WPA, on the other hand, was associated with lower cardio-respiratory fitness and not mortality. In addition, we report that LTPA and SPA are not related to WPA. Based on these findings one may speculate that only physical activity which increases cardio-respiratory fitness is associated with lower mortality. We acknowledge that only a subgroup of the total study population participated in CPET and that the mortality of the sensitivity analysis in this group resulted in non-significant findings (most likely due to the low number of cases). Hence, further research is required to untangle the relationship between domain specific physical activity and cardio-respiratory fitness with regards to mortality.

Even though, SHIP and CARLA comprise population-based and methodologically rigorous regionally representative surveys of Germany, the findings of our analyses need to be interpreted in the context of several limitations. First, our analysis consisted of white Europeans living in rural areas. Therefore, we do not know whether our findings are also applicable to other ethnicities. Second, we used only one time point to define physical activity behavior. Hence, we do not know whether changes in LTPA, SPA and WPA are associated with mortality or cardio-respiratory fitness. Third, although we used a directed acyclic graph to identify metabolic and cardiovascular confounders for our multivariable models, we cannot exclude the possibility of further residual confounding. Fourth, the analyses of CVD and cancer mortality in SHIP may be limited by reduced statistical power due to a low number of events (n = 106 and n = 84, respectively). Fifth, because of the higher age in CARLA only a small proportion (22%) of participants were employed at time of examination which especially could have hampered the WPA analysis. Sixth, the Baecke questionnaire does not quantify sedentarism, which is independently associated with an increased risk for non-communicable diseases like CVD and type 2 diabetes mellitus. Future studies should use objective measures of inactivity in different domains of physical activity to assess whether the results of this report are sustained. Irrespective of these limitations, strengths of our study are the population-based setting, the two independent studies, the use of standardized data collection methods, the capacity to perform adjustment for a variety of clinical risk factors and the availability of CPET results from 1,493 participants.

The main findings of this study are threefold. First, our results support previous findings that higher levels of LTPA and SPA are associated with a lower mortality risk. Second, our results suggest no protective or adverse association of WPA with mortality. Third, in a subset of the study population WPA was not associated with cardio-respiratory fitness which may elude to the hypothesis that only physical activity which increases cardio-respiratory fitness is protective from mortality. This adds important information to the ongoing discussion regarding the impact of domain specific physical activity on mortality.

LTPA and SPA, but not WPA, are related to higher cardio-respiratory fitness and a lower mortality risk. The results of this study suggest that the beneficial effects of exercise may be driven by higher cardio-respiratory fitness. Since current guidelines recommend physical activity independent of the domain in which it occurs, our findings suggest that LTPA and SPA should be prioritized. However, further studies with objectively measured WPA and CPET are warranted.

## Methods

The Study of Health in Pomerania (SHIP) is a prospective population-based cohort of adults from West Pomerania, a north-eastern region in Germany of approximately 220,000 inhabitants. The first sample (SHIP-0) was surveyed between 1997 and 2001 using a stratified cluster-random sample of 7,008 individuals. The net sample (without migrated or deceased persons) included 6,265 eligible individuals. A total of 4,308 (2,192 women) subjects participated (response: 68.8%) in SHIP-0^34^. All participants of the first examination cycle were invited for a follow-up visit between 2002 and 2006 (SHIP-1). Measurements of self-reported physical activity and CPET were undertaken during the SHIP-1 examination, which is why we defined the SHIP-1 examination cycle as the baseline examination for the present analyses. A total of 3,300 subjects participated in SHIP-1 (age range: 25–86; 48% male). Individuals with prevalent myocardial infarction, stroke or cancer as well as those who died within the first year of follow-up were excluded from this analysis.

The Cardiovascular Disease, Living and Ageing in Halle study (CARLA) is a cohort study of a representative sample of the citizens of the city of Halle in eastern Germany with a total of 1,779 participants (age range 45–83 years of age at baseline; 812 women). Details of the study design and methods have been described elsewhere^35^. The baseline examination took place between December 2002 and January 2006.

All-cause and cause-specific mortality was determined after a median follow-up of 8.2 years (25^th^ and 75^th^ percentile: 7.4 and 9.2) in SHIP and 11.5 years (25^th^ and 75^th^ percentile: 10.7–12.4) in CARLA. All participants provided written informed consent. SHIP and CARLA were approved by the Ethics Committee of the University Medicine Greifswald and Halle, respectively. Further, all experiments and examinations were performed in accordance with relevant guidelines and regulations.

### Interview, medical and laboratory examination

In SHIP and CARLA data were collected with respect to the participant’s socio-economic characteristics, physical activity and health status using a standardized computer-assisted personal interviews, medical examinations by certified personnel, and laboratory measurements. Height and weight of the subjects were measured. Body mass index (BMI) was calculated by dividing body height [m] by body mass [kg] squared. Smoking status was assessed by questionnaire (SHIP) or during a standardized interview (CARLA).

Vital status information of study participants was regularly collected from population registries. Participants were censored at loss to follow-up. Death certificates were requested from the local health authorities and were coded by a certified nosologist according to the International Classification of Diseases, 10^th^ revision (ICD-10). Furthermore, in SHIP two internists (S.G. & M.D.) independently validated the underlying cause of death and performed a joint reading together with a third internist (H.V.) in cases of disagreement^36^. CVD mortality was defined as ICD-10: I10-I79, R96. Cancer mortality was defined as ICD-10: C00-C97.

### Physical activity assessment

Domain specific physical activity was assessed using the well-established Baecke questionnaire^37^. This questionnaire evaluated the level of domain specific physical activity^38,39^. Briefly, the questionnaire consists of 16 questions in three distinct sections: physical activity during leisure time excluding sport (i.e. one’s own LTPA compared to others of similar age as well as sweating, playing sports, watching television, walking and cycling during leisure time), SPA (i.e. identification of the sports played followed by questions regarding duration per week and months per year) and WPA (i.e. one’s own WPA compared to others of similar age followed by questions regarding sitting, standing, walking, lifting of heavy loads and sweating at work as well as if one is tired after work). Most questions are scored on a five-point Likert scale, ranging from never to always or very often. For the reported sport activities, additional questions query the number of months per year and hours per week of participation. The three derived indices, LTPA, SPA and WPA, are scored in arbitrary units ranging from 1 to 5. The Baecke questionnaire has been used in a number of studies in various populations and its validity as well as reliability have been thoroughly tested (interclass correlation from 0.65 to 0.92 and a Pearson correlation with doubly labeled water of 0.54 to 0.69)^37–42^.

### Symptom-limited cardiopulmonary exercise testing to determine cardiorespiratory fitness

In SHIP participants were invited to participate in CPET according to a modified Jones protocol using a calibrated electromagnetically braked cycle ergometer (Ergoselect 100, Ergoline, Germany). After 3 minutes of unloaded cycling plus the ergometer related permanent load, work load increased step-wise at a rate of 16 Watts/min^43^. Gas exchange and ventilatory variables were analyzed breath by breath averaged over 10-second intervals using a computer based system^44^. In the absence of chest pain and ECG abnormalities, all tests were continued as symptom-limited (volitional exertion, dyspnea or fatigue). Prior to the test, patients were encouraged to reach maximal exhaustion, while during exercise no further motivational interventions were utilized. All tests were performed at room air according to current guidelines for exercise testing, with continuous monitoring of ECG, blood pressure and oxygen saturation^45–47^. VO_2_peak was defined as the highest 10-second average of VO_2_ in the last minute of exercise. The anaerobic threshold (VO_2_@AT) was based on non-invasive determination by gas exchange analysis by assessing the relation of VO_2_ to VCO_2_ (V-slope method)^48^. Wmax was characterized as the highest reached power on the bicycle ergometer during exercise kept for at least 20 s at VO_2_peak.

### Statistical analysis

Data on quantitative characteristics are expressed as median (25^th^ and 75^th^ percentile). Data on categorical characteristics are expressed as absolute numbers and percent values. Multivariable Cox proportional hazards regression analysis was used to associate the Baecke indices and CPET parameters with all-cause, CVD, and cancer mortality (each outcome considered separately). Pearson correlation coefficients (r) were used to assess the relation between LTPA, SPA and WPA. For the mortality analysis subjects who died within the first year of follow-up were excluded and the number of years between birth and death or censoring served as the time axis^49^. Hazard ratios (HR) and 95% confidence intervals (CI) were estimated. The assumption of proportionality of hazards was confirmed by assessing the parallelism of survival curves of log-log plots and by Schoenfeld residuals. The assumption of linearity of the log-hazards underlying the Cox model was investigated using multivariable fractional polynomials^50^, indicating that this assumption was not violated for the continuous exposure variables. Therefore, we reported HRs and linear regression coefficients per standard deviation increase. We also performed a sensitivity analysis with regards to the association of the Baecke indices and all-cause, CVD as well as cancer mortality in subjects who participated in CPET.

We selected covariates based on a directed acyclic graph for parameters known to affect physical activity and cardio-respiratory fitness as well as mortality. Specifically, the models were adjusted for sex, age, smoking, alcohol consumption, years of schooling, income, body weight and height. Income was ‘equalized’ by dividing the household income (in €) by the square root of the number of household members. Smoking status was categorized in never, ex-smoker, or current smoker and alcohol consumption (in grams per day) was derived from a beverage-specific quantity-frequency index.

Linear regression analysis was used to assess the relation between LTPA, SPA and WPA with VO_2_peak and VO_2_peak/kg. Subjects with asthma or chronic lung disease were excluded from this analysis. These models were adjusted for age, sex, body height, body weight and current smoking status. Results of this analysis are presented as linear regression coefficient (β) and 95%-CI. All statistical analyses were performed using Stata 13.1 (Stata Corporation, College Station, TX, U.S.A.) and SAS 9.4 (SAS Institute Inc., Cary, NC, USA).

### Ethics approval and consent to participate

All participants provided written informed consent and SHIP as well as CARLA were approved by the Ethics Committee of the University Medicine Greifswald and Halle, respectively.

## Electronic supplementary material

Supplementary Information

## Data Availability

S.H.I.P. and C.A.R.L.A. data are publically available and can be applied for at www.community-medicine.de and http://www.medizin.uni-halle.de/index.php?id=1109, respectively.
