# Supplementary material for: Association of domain-specific physical activity and cardiorespiratory fitness with all-cause and cause-specific mortality in two population-based cohort studies
Source: Sci Rep. 2018 Oct 30;8:16066. doi: 10.1038/s41598-018-34468-7 (PMC6207740; doi:10.1038/s41598-018-34468-7)
Supplement: Supplementary file 1 — Supplementary Information [file 41598_2018_34468_MOESM1_ESM.pdf]

Suppl. information for

Title: Association of domain-specific physical activity and cardiorespiratory fitness with all-cause and cause-specific mortality in two population-based cohort studies

Authors: Martin Bahls, Ph.D. *[1,2], Stefan Groß, Ph.D.[1,2], Sebastian E. Baumeister, Ph.D.[3], Henry Völzke, M.D. [2,4], Sven Gläser, M.D. [5], Ralf Ewert, M.D. [1,2], Marcello R.P. Markus, M.D. [1,2], Daniel Medenwald, M.D.[6], Alexander Kluttig, Ph.D.[6], Stephan B. Felix, M.D. [1,2], Marcus Dörr, M.D. [1,2]

Suppl. Table 1 - Population descriptives of the SHIP study participants who died of CVD. All values are given as median (25th and 75th percentile) or percentage for categorical variables. BMI: body mass index. VO_2peak: peak oxygen consumption. VO_2@AT: oxygen consumption at the aerobic threshold. LTPA: leisure time physical activity. SPA: sports-related physical activity. WPA: work-related physical activity.

Parameter		Survivers only (n = 2769)	CVD mortality subgroup (n = 113)	All subjects (n = 2882)
Age (years)		52 (40; 64)	75 (69; 81)	53 (41; 65)
Income (€)		1100 (778; 1525)	1100 (779; 1450)	1100 (778; 1525)
alcohol consumption (ml/day)		3.9 (1.14; 12.13)	1.31 (0; 10.29)	3.93 (1.01 (12.12)
School education, % <10 years		35.5	80.0	37.2
BMI (kg/m2)		27 (24; 31)	29 (27; 33)	27 (24; 31)
VO_2peak (ml/min)		1900 (1534; 2383)	1385 (1010; 1713)	1884 (1524; 2378)
VO_2@AT (ml/min)		1050 (900; 1300)	950 (750; 1200)	1050 (900; 1300)
max. Watt		148 (116; 180	100 (68; 116)	148 (116; 180)
LTPA		3.25 (2.75; 3.75)	3 (2.5; 3.5)	3.25 (2.75; 3.75)
SPA		2.25 (2.00; 2.75)	2 (1.75; 2.5)	2.25 (2; 2.75)
WPA		2.86 (2.14; 3.57)	3 (2.29; 3.71)	2.86 (2.14; 3.57)
Sex (%male)		47	67	47
Smoke status	never	26.9	15	26.5
	former	42.2	33.6	41.9
	current	30.8	51.33	31.6

Suppl. Table 2 - Population descriptives of the SHIP study participants who died of cancer. All values are given as median (25th and 75th percentile) or percentage for categorical variables. BMI: body mass index. VO_2peak: peak oxygen consumption. VO_2@AT: oxygen consumption at the aerobic threshold. LTPA: leisure time physical activity. SPA: sports-related physical activity. WPA: work-related physical activity.

Parameter		Survivers only (n = 2797)	Cancer mortality subgroup (n = 85)	All subjects (n = 2882)
Age (years)		52 (40; 64)	69 (60; 76)	53 (41; 65)
Income (€)		1100 (778; 1525)	1096 (778; 1450)	1100 (778; 1525)
alcohol consumption (ml/day)		3.9 (1.02; 12.12)	3.45 (0.65; 11.50)	3.93 (1.01; 12.12)
School education, % <10 years		36.2	72.9	37.23
BMI (kg/m2)		27 (24; 31)	29 (26; 32)	27 (24; 31)
VO2peak (ml/min)		1900 (1526; 2395)	1654 (1436; 1900)	1884 (1524; 2378)
VO2@AT (ml/min)		1050 (900; 1300)	1000 (900; 1100)	1050 (900; 1300)
max. Watt		148 (116; 180	116 (100; 148)	148 (116; 180)
LTPA		3.25 (2.75; 3.75)	3.25 (2.63; 3.63)	3.25 (2.75; 3.75)
SPA		2.25 (2.00; 2.75)	2 (1.75; 2.5)	2.25 (2; 2.75)
WPA		2.86 (2.14; 3.57)	3 (2.57; 3.29)	2.86 (2.14; 3.57)
Sex (%male)		47	72	47
Smoke status	never	26.6	23.5	26.5
	former	42.3	27.1	41.9
	current	31.1	49.4	31.6

Suppl. Table 3 Characteristics of participants according to physical activity domain categories in SHIP-1. Q1-Q4: quartiles 1 to 4. Entries are median (25th, 75th percentile) or %. P-trend from Cuzick's non-parametric test. BMI: body mass index. VO_2peak: peak oxygen consumption. VO_2@AT: oxygen consumption at the aerobic threshold. LTPA: leisure time physical activity. SPA: sports-related physical activity. WPA: work-related physical activity.

	Quartiles of the Baecke questionnaire indices				
	Q1	Q2	Q3	Q4	P (linear trend)
			**LTPA (n = 2,911)**		
N (min – max)	837 (1.0 - 2.8)	854 (3.0 - 3.3)	781 (3.5 - 3.8)	439 (4.0 - 5.0)	
Female, %	50.4	50.0	54.3	57.4	0.006
Age, years	52 (40, 66)	54 (42, 65)	54 (41, 64)	52 (40, 63)	0.126
School education, % <10 years	38.6	38.3	37.8	33.7	0.087
Income, €	1,100 (778, 1472)	1,100 (895, 1,472)	1,100 (894, 1,550)	1,140 (778, 1,525)	0.287
Smoking status, %					0.001
Never	36.1	41.6	44.8	46.9	
Former	30.8	27.1	23.8	23.1	
Current	33.1	31.3	31.4	30.1	
Alcohol consumption, gr/day	3.9 (0.7, 12.8)	4.3 (1.3, 13.1)	3.9 (1.1, 11.2)	3.5 (1.1, 10.7)	0.723
BMI, kg/m²	28.1 (24.7, 31.6)	27.6 (24.5, 31.0)	27.1 (24.4, 30.1)	26.3 (23.7, 29.8)	<0.001
VO_2peak (ml/min)	1,850 (1,500, 2,350)	1,858 (1,500, 2,409)	1,900 (1,550, 2,400)	1,906 (1,593, 2,328)	0.154
VO_2@AT (ml/min)	1,000 (850, 1,250)	1,050 (900, 1,250)	1,100 (900, 1,330)	1,100 (900, 1,300)	0.002
max. Watt	148 (116, 180)	148 (116, 180)	148 (116, 196)	148 (116, 180)	0.020
			**SPA (n = 2,910)**		
n (min – max)	1,150 (1.0 -2.0)	415 (2.2 - 2.3)	631 (2.5 – 2.8)	714 (3.0 – 5.0)	
Female, %	51.7	50.8	58.5	48.9	0.958
Age, years	55 (42, 67)	53 (42, 64)	53 (40, 63)	51 (39, 63)	<0.001
School education, % <10 years	46.3	39.0	34.4	25.4	<0.001

Income, €	1,096 (778, 1,450)	1,100 (778, 1,472)	1,184 (895, 1,550)	1,450 (1,096, 1,760)	<0.001
Smoking status, %					<0.001
Never	38.9	39.0	45.0	44.8	
Former	29.4	30.6	23.6	22.8	
Current	31.7	30.4	31.4	32.5	
Alcohol consumption, gr/day	3.2 (0.7, 11.0)	4.0 (1.1, 13.4)	3.9 (1.2, 11.2)	5.2 (1.3, 13.5)	<0.001
BMI, kg/m²	28.0 (24.9, 31.6)	27.6 (24.8, 30.8)	27.4 (24.1, 30.9)	26.2 (23.7, 29.4)	<0.001
VO_2peak (ml/min)	1,750 (1,450, 2,207)	1,954 (1,600, 2,328)	1,815 (1,510, 2,296)	2,050 (1,648, 2,600)	<0.001
VO_2@AT (ml/min)	1,000 (850, 1,200)	1,100 (900, 1,250)	1,050 (900, 1,300)	1,150 (950, 1,400)	<0.001
max. Watt	132 (116, 180)	148 (132, 180)	148 (116, 180)	164 (132, 212)	<0.001
			**WPA (n = 1,434)**		
n (min – max)	365 (1 - 2.1)	424 (2.3 - 2.9)	355 (3.0 – 3.6)	290 (3.7 – 4.9)	
Female, %	57.3	55.7	49.0	48.3	0.006
Age, years	44 (37, 52)	45 (38, 54)	44 (36, 52)	43 (35, 49)	0.008
School education, % <10 years	6.3	10.9	14.9	23.1	<0.001
Income, €	1,550 (1,275, 1,900)	1,472 (1,096, 1,803)	1,183 (895, 1,525)	1,096 (778, 1,450)	<0.001
Smoking status, %					<0.001
Never	43.3	44.1	38.3	30.3	
Former	25.6	27.4	36.6	43.8	
Current	27.1	28.5	25.1	25.9	
Alcohol consumption, gr/day	5.6 (2.0, 14.9)	6.7 (2.1, 15.7)	5.0 (1.3, 13.3)	5.4 (1.7, 14.3)	0.440
BMI, kg/m²	26.0 (23.1, 29.1)	26.4 (23.3, 30.2)	26.1 (23.5, 29.5)	26.6 (24.1, 29.7)	0.198
VO_2peak (ml/min)	2,049 (1,609, 2,500)	2,017 (1,619, 2,598)	2,128 (1,660, 2,542)	2,100 (1,691, 2,609)	0.261
VO_2@AT (ml/min)	1,100 (900, 1,300)	1,150 (950, 1,400)	1,100 (910, 1,335)	1,100 (950, 1,300)	0.707
max. Watt	164 (132, 196)	164 (132, 196)	164 (132, 212)	164 (132, 196)	0.697

Suppl. Table 4 - Population descriptives of the CARLA study participants who died of CVD. All values are given as median (25th and 75th percentile) or percentage for categorical variables. BMI: body mass index. VO_2peak: peak oxygen consumption. VO_2@AT: oxygen consumption at the aerobic threshold. LTPA: leisure time physical activity. SPA: sports-related physical activity. WPA: work-related physical activity.

Parameter		Survivers only (n = 1362)	CVD mortality subgroup (n = 157)	All subjects (n = 1519)
Age (years)		61.5 (54.0; 68.5)	77.1 (70.4; 79.6)	62.6 (54.7; 70.5)
Income (€)		1750 (1250; 2250)	1750 (1250; 1750)	1750 (1250; 2250)
alcohol consumption (ml/day)		5.0 (0.0; 16.8)	2.7 (0.0; 13.8)	5.0 (0.0; 16.2)
School education, % <10 years		34.2	65.0	37.35
BMI (kg/m^2)		27.8 (25.0; 30.9)	27.9 (25.2; 31.6)	27.8 (25.0; 31.0)
LTPA		3.3 (2.8; 3.5)	3.0 (2.5; 3.3)	3.3 (2.8; 3.5)
SPA		2.3 (1.8; 3.0)	2.0 (1.5; 2.5)	2.3 (1.8; 3.0)
WPA		2.6 (2.1; 3.4)	2.9 (2.5; 3.3)	2.6 (2.1; 3.4)
Sex (%male)		49.8	66.2	51.5
Smoke status	never	47.9	38.8	46.9
	former	32.6	47.1	34.1
	current	19.5	14.0	19.0

Suppl. Table 5 - Population descriptives of the CARLA study participants who died of cancer. All values are given as median (25th and 75th percentile) or percentage for categorical variables. BMI: body mass index. VO_2peak: peak oxygen consumption. VO_2@AT: oxygen consumption at the aerobic threshold. LTPA: leisure time physical activity. SPA: sports-related physical activity. WPA: work-related physical activity.

Parameter		Survivers only (n = 1362)	Cancer mortality subgroup (n = 136)	All subjects (n = 1495)
Age (years)		61.5 (54.0; 68.5)	72.6 (65.4; 77.9)	62.6 (54.5; 69.6)
Income (€)		1750 (1250; 2250)	1750 (1250; 2250)	1750 (1250; 2250)
alcohol consumption (ml/day)		5.0 (0.0; 16.8)	5.4 (0.0; 23.0)	5.0 (0.0; 17.4)
School education, % <10 years		34.2	61.3	36.56
BMI (kg/m2)		27.8 (25.0; 30.9)	28.4 (25.5; 30.9)	27.8 (25.0; 30.9)
LTPA		3.3 (2.8; 3.5)	3.0 (2.5; 3.5)	3.3 (2.8; 3.5)
SPA		2.3 (1.8; 3.0)	2.0 (1.8; 2.8)	2.3 (1.8; 3.0)
WPA		2.6 (2.1; 3.4)	3.5 (2.3; 3.9)	2.6 (2.1; 3.4)
Sex (%male)		49.8	71.5	51.8
Smoke status	never	47.9	33.1	46.5
	former	32.6	45.6	33.8
	current	19.5	21.3	19.7

Suppl. Table 6 Characteristics of participants according to physical activity domain categories in CARLA. Q1-Q4: quartiles 1 to 4. Entries are median (25th, 75th percentile) or %. P-trend from Cuzick's non-parametric test.BMI: body mass index. LTPA: leisure time physical activity. SPA: sports-related physical activity. WPA: work-related physical activity.

	Quartiles of the Baecke questionnaire indices				
	Q1	Q2	Q3	Q4	P (linear trend)
**LTPA (N = 1,776)**					
N (min – max)	346 (1.2-2.5)	484 (2.7-3.0)	555 (3.2-3.5)	391 (3.7-5.0)	
Female, %	42	45	41	33	<0.001
Age, years	65 (56, 75)	65 (56, 75)	64 (55, 74)	62 (55, 68)	0.13
School education, % <10 years	45	42	37	36	<0.001
Income, €	1,750 (1,250, 2,250)	1,750 (1,250, 2,250)	1,750 (1,250, 2,250)	1,750 (1,250, 2,250)	<0.001
Smoking status, %					<0.001
Never	35.8	50.2	45.8	44.8	
Former	38.4	31.6	36.9	37.1	
Current	25.7	18.2	17.3	18.2	
Alcohol consumption, gr/day	5.0 (0, 2.9)	5.0 (0, 18.9)	5.0 (0, 15.6)	5.0 (0, 16.2)	<0.001
BMI, kg/m²	29.0 (26.2, 32.5)	27.9 (25.4, 30.8)	27.7 (25.0, 30.6)	27.1 (24.3, 30.1)	0.85
**SPA (N =1,770)**					
n (min – max)	508 (1.0-1.7)	473 (2-2.2)	334 (2.5-2.7)	455 (3.0-5.0)	
Female, %	46.85	42.49	45.21	47.91	<0.001
Age, years	64.51 (54.48, 75.14)	63.55 (54.94, 73.03)	64.3 (57.69, 72.32)	64.29 (56.36, 70.38)	<0.001
School education, % <10 years	45.47	41.53	37.43	36.48	<0.001
Income, €	1,750 (1,250, 2,250)	1,750 (1,250, 2,250)	1,750 (1,250, 2,250)	1,750 (1,250, 2,250)	<0.001
Smoking status, %					<0.001
Never	41.9	40.6	47.6	50.5	

Former	33.7	35.9	39.8	35.6	
Current	24.4	23.5	12.6	13.8	
Alcohol consumption, gr/day	2.7 (0.0, 16.5)	5.0 (0.0, 16.2)	5.0 (0, 16.2)	7.4 (0.0, 18.9)	<0.001
BMI, kg/m²	27.7 (25.0, 31.0)	28.0 (25.5, 31.1)	28.1 (25.4, 31.4)	27.7 (24.7, 30.1)	<0.001
			**WPA (N =392)**		
n (min – max)	88 (1.2-2.0)	96 (2.1-2.5)	108 (2.6-3.2)	100 (3.4-4.5)	
Female, %	53.41	38.54	37.96	25.00	<0.001
Age, years	52.97 (50.67, 57.87)	53.64 (50.18, 57.15)	53.44 (51.16, 56.53)	53.41 (49.25, 56.54)	0.28
School education, % <10 years	9.09	5.21	9.26	9.00	0.63
Income, €	2,750 (1,750, 3,250)	2,750 (2,250, 3,250)	2,250 (1,750, 2,750)	2,250 (1,750, 2,750)	<0.001
Smoking status, %					<0.001
Never	46.6	50.0	36.1	32.0	
Former	28.4	32.3	31.5	35.0	
Current	25.0	17.7	32.4	33.0	
Alcohol consumption, gr/day	4.7 (0.0, 13.4)	7.6 (2.5, 22.4)	10.8 (2.8, 24.5)	13.3 (4.5, 29.6)	<0.001
BMI, kg/m²	27.7 (25.0, 31.1)	28.0 (25.4, 31.1)	28.1 (25.4, 31.4)	27.7 (24.7, 30.1)	0.67

Suppl. Table 7 Hazards ratios for the association between physical activity domains with all-cause and cause-specific mortality in SHIP participants who also completed the CPET. PY: person years. CI: confidence interval. CVD: cardiovascular disease. LTPA: leisure time physical activity. SPA: sports related physical activity. WPA: work related physical activity. CVD mortality was defined as ICD-10: I10-I79, R96. Cancer mortality was defined as ICD-10: C00-C97.

	all-cause			CVD mortality			Cancer mortality		
	cases/person years	incidence per 10000 PY	HR (95% CI)	cases/person years	incidence per 10000 PY	HR (95% CI)	cases/person years	incidence per 10000 PY	HR (95% CI)
LTPA	80/12416	64	0.91 (0.72 to 1.17)	18/12416	14	0.76 (0.44 to 1.27)	30/12357	24	1.02 (0.68 to 1.52)
SPA	80/12349	65	0.94 (0.75 to 1.18)	18/12349	15	1.01 (0.63 to 1.62)	30/12349	24	**0.65 (0.43 to 0.99)**
WPA	20/6975	29	0.83 (0.50 to 1.38)	4/6975	6	0.99 (0.27 to 3.76)	7/6974	10	0.62 (0.28 to 1.37)
